# UBC Mediated by SEPT6 Inhibited the Progression of Prostate Cancer

**DOI:** 10.1155/2021/7393029

**Published:** 2021-12-20

**Authors:** Ruochen Zhang, Yaojing Yang, Haijian Huang, Tao Li, Liefu Ye, Le Lin, Yongbao Wei

**Affiliations:** Shengli Clinical Medical College of Fujian Medical University, Department of Urology, Fujian Provincial Hospital, China

## Abstract

**Background:**

Prostate cancer is one of the most common malignancies in men. Protein ubiquitination is an important mechanism for regulating protein activity and level *in vivo*. We aimed to study the mechanism of SEPT6 and UBC action in prostate cancer to identify new targets.

**Methods:**

The ubiquitin-protein and the ubiquitin coding gene UBA52, UBA80, UBB, and UBC expressions were detected in clinical tissues and cells. Overexpression and knockdown of UBC were performed in prostate cancer DU145 cells. Cell Counting Kit 8 (CCK-8) assay was performed to detect cell proliferation. Cell cycle at 24 h was detected by flow cytometry. Clonal formation assay was used to measure cell clone number. Immunofluorescence (IF) was performed to detect the colocalization of SEPT6 and UBC in prostate cancer cells. Next, we overexpressed or knocked down SEPT6 expression in DU145 cells. Pearson correlation coefficient was applied to analyze the relationship between SEPT6 and UBC in prostate cancer tissue. oe-SEPT6+oe-UBC coexpressing cells were constructed to detect the upstream and downstream relationship between SEPT6 and UBC on prostate cancer cells. The tumor formation experiment was performed to explore SEPT6/UBC effect on prostate cancer.

**Results:**

UBC was upregulated in prostate cancer tissues and cells. Overexpression of UBC promoted cell survival and proliferation. IF revealed the colocalization of SEPT6 and UBC in prostate cancer cells. UBC expression decreased after oe-SEPT6, while increased after sh-SEPT6, indicating that UBC was downstream of SEPT6. Pearson correlation coefficient analysis showed that SEPT6 was negatively correlated with UBC in prostate cancer tissues. SEPT6 as an upstream gene of UBC regulated prostate cancer cell behavior through UBC. The tumor formation experiment showed that SEPT6 could inhibit tumor growth.

**Conclusion:**

In general, SEPT6 inhibited UBC expression, thereby reducing the overall ubiquitination level, affecting the expression level of downstream cell proliferation-related genes, and then affecting the progression of prostate cancer.

## 1. Introduction

Prostate cancer is one of the most common malignancies and the second most common cause of cancer-related death in men [1]. It is a complex, heterogeneous disease with a variable natural history [2]. Prostate cancer is characterized by various biological behaviors translated into different clinical manifestations, ranging from inert microscopic disease to highly aggressive tumors, including a tendency to metastasize [3]. There are three recognized risk factors for prostate cancer: age, race, and genetics [1]. The key problem for prostate cancer patients is detecting recurrent disease and treating metastatic cancer [4]. Prostate-specific antigen (PSA) targeting strategy improved functional imaging can enhance the identification of patients with less metastatic prostate cancer in the short term [5]. However, the diagnostic method of PSA screening for prostate cancer in people has always been controversial and fiercely debated, which is associated with overdiagnosis and overtreatment, accompanied by urinary, sexual, and intestinal dysfunction [6]. Despite the new progress, prostate cancer remains a major medical problem affecting men. Current trends in the treatment of prostate cancer patients increase the need for reliable therapeutic targets and new and innovative strategies for cancer diagnosis and treatment.

Septins are a class of guanosine triphosphate- (GTP-) binding proteins that are highly conserved in eukaryotes and are usually associated with membranes and are involved in cytokinesis, exocytosis, and other cellular processes [7, 8]. Human Septins contain 13 gene families that encode more than 30 protein isotypes with ubiquitous and tissue-specific expression [9]. Septins are involved in various normal cellular processes and may be a key cancer gene and participate in the pathogenesis of multiple diseases, including cancer [10, 11]. Septin6 (SEPT6), also known as SEP2 and KIAA0128, is located in XQ24 [12]. It plays a role in actin dynamics, cell shape, and microtubule regulation [13]. SEPT6 and SEPT7 GTP-binding proteins regulate AP3 and endosomal-sorting complex required for transport- (ESCRT-) dependent multivesicle biogenesis [14]. SEPT6 is involved in the development of hepatocellular carcinoma (HCC) [15] and also plays a role in the occurrence and normal function of leukemia (including neurotransmission) [16]. A previous study has found that SEPT6 is a target gene of microRNA-223-3p (miR-223-3p), which may reverse the biological activity induced by miR-223-3p and may provide a potential therapeutic target for prostate cancer [17]. This suggests that SEPT6 may be a potential therapeutic target.

Ubiquitin is a highly conserved 76 amino acid protein in all eukaryotes [18]. Ubiquitination is catalyzed by a three-enzyme cascade reaction composed of E1 ubiquitin-activating enzyme, E2 ubiquitin-conjugating enzyme, and E3 ubiquitin ligase [19]. Through the action of ubiquitin-protein ligase, ubiquitin is connected to the target protein through the isopeptide bond between the C-terminal glycine residue of ubiquitin and the epsilon amino group of lysine in the substrate protein [20]. Like phosphorylation, ubiquitination of proteins regulates or affects most cellular processes, and defects in this system are associated with many diseases [21]. Ubiquitin C (UBC) is one of the two stress-regulated polyubiquitin genes in mammals and plays a crucial role in maintaining cellular ubiquitin levels, especially under stress conditions [22]. Studies have shown that UBC is an essential source of ubiquitin in the process of cell proliferation and stress and is necessary for fetal liver development, cell cycle progression, and stress tolerance [23]. Ubiquitin-mediated proteasome degradation is an important mechanism for regulating protein metabolism in cells and is of great significance for maintaining normal functions of the body [24]. Ubiquitin proteasome system (UPS) has made some progress in developing potential novel therapies against cancer and neurodegenerative diseases [25, 26], but the specific mechanism of UBC in prostate cancer is rarely studied.

Based on the above studies, the antitumor effect of SEPT6 in prostate cancer may be used as a target for the development of prostate cancer disease. SEPT6 may target the UBC gene to affect prostate cancer, but its effect on prostate cancer cells remains unknown. Therefore, this study is aimed at exploring the role of the expression of SEPT6 and UBC in prostate cancer tissues and cells and at providing new insights for the development of prostate cancer therapy.

## 2. Material and Methods

### 2.1. Clinical Tissue Samples

Ten participants came from Fujian Provincial Hospital. Prostate cancer tissues and paracancerous tissues were collected and divided into prostate cancer tissue and paracancerous tissue groups. All subjects gave their informed consent for inclusion before they participated in the study. The study was conducted following the Declaration of Helsinki; written informed consents were obtained from the guardians of these patients.

### 2.2. Cell Culture and Treatment

Prostate cell lines RWPE-1 and prostate cancer cell lines PC3, CWR22 (22Rv1), VCaP, and DU145 were purchased from the Cell Bank of the Chinese Academy of Sciences. RWPE-1 and VCaP cells were cultured in Dulbecco's Modified Eagle Medium-High Glucose (DMEM-H) containing 10% fetal bovine serum (FBS, Thermo Fisher Scientific). PC3 cells were cultured in Ham's F-12K (Kaighn's) with 10% FBS. CWR22 and DU145 cells were cultured in Roswell Park Memorial Institute (RPMI) 1640 (w/o Hepes) containing 10% FBS. To overexpress UBC and SEPT6, the UBC and SEPT6 sequences were linked to the vector LV003. According to the instructions, UBC and SEPT6 vectors were transfected into DU145 cells using Lipofectamine 3000 reagent. The oe-NC group was transfected with an oe-NC plasmid in DU145 cells. The short hairpin targeting UBC including sh-UBC (1), sh-UBC (2), and sh-UBC (3) and the short hairpin targeting SEPT6 including sh-SEPT6 (1), sh-SEPT6 (2), and sh-SEPT6 (3) synthesized by Sangon Biotech Co., Ltd. (Shanghai, China), and the corresponding negative control sh-NC were used to knock down the expression of UBC and SEPT6. To detect the relationship between the upstream and downstream effects of SEPT6 and UBC on prostate cancer cells, an oe-SEPT6+oe-UBC coexpression cell line was constructed.

### 2.3. Quantitative Real-Time PCR (qRT-PCR)

qRT-PCR was used to detect the mRNA levels of UBA52, UBA80, UBB, UBC, SEPT6, CDK1, CCNA2, MCM10, E2F1, HIST1H1A, HIST1H3B, BRCA1, and AURKB in cells and tissues. Total RNA was extracted by Trizol methods. RNA was reversely transcribed into cDNAs by the instruction of a reverse transcription kit (CW2569, CWBIO, China). SYBR Green qPCR mix (Invitrogen) was performed to test gene's relative expression in ABI 7900 system. The relative level of the gene was calculated by the 2^-*ΔΔ*Ct^ method with GAPDH as the internal gene. The primer sequences used in this study are shown in Table 1.

### 2.4. Western Blot (WB)

RIPA lysis buffer (#P0013B, Beyotime Biotechnology) was applied to extract the total protein from prostate cancer tissues and cells. The protein of each group was quantified according to the BCA protein determination kit. The protein was mixed with SDS-PAGE loading buffer (#MB2479, Meilunbio) for 5 min in boiling water at 100°C. The protein was adsorbed on the PVDF membrane by gel electrophoresis, sealed with 5% skim milk solution for 2 h at room temperature, and incubated with diluted primary antibodies at room temperature for 90 minutes. The secondary antibody HRP goat anti-mouse IgG (SA00001-1, 1 : 5000, Proteintech, USA) and HRP goat anti-Rabbit IgG (SA00001-2, 1 : 6000, Proteintech, USA) were incubated with the membrane at room temperature for 90 min. The protein bands were detected by the ChemiScope 6100 system (Clinx Co., Ltd, Shanghai, China). *β*-Actin was used as the internal reference for detecting relative expression levels. The primary antibodies were SEPT6 (ab138036, 1 : 750, Abcam, UK), UBA52 (18039-1-AP, 1 : 3000, Proteintech), UBA80 (14946-1-AP, 1 : 750, Proteintech, USA), UBB (10201-2-AP, 1 : 1000, Proteintech, USA), UBC (14415-1-AP, 1 : 2000, Proteintech, USA), ubiquitin (ab7780, 1: 2000, Abcam, UK), Ki67 (ab92742, 1 : 5000, Abcam, UK), PCNA (10205-2-AP, 1 : 3000, Proteintech, USA), and *β*-actin (66009-1-Ig, 1 : 5000, Proteintech, USA).

### 2.5. Immunohistochemistry (IHC) and Immunocytochemistry (ICC)

Ubiquitin-protein expression was detected by IHC in prostate cancer tissues and paracancerous tissues. The slices were roasted at 60°C for 12 h. Then, the slices were dewaxed to water and heated to repair the antigen. 1% periodic acid was added, and the endogenous enzyme was inactivated for 10 min at room temperature. PBS was washed 3 times for 3 min. Ubiquitin (ab7780, 1 : 100, Abcam, UK) primary antibody was incubated overnight at 4°C, and PBS was washed 3 times for 5 min. The expressions of ubiquitin in prostate cell RWPE-1 and prostate cancer cells PC3, CWR22, VCAP, and DU145 were detected by ICC. The slides were removed and fixed with 4% paraformaldehyde for 30 min. PBS was washed 3 times for 5 min. Then, 0.3% Trolaton was added for 30 min at 37°C. 3% H_2_O_2_ was added at room temperature for 10 min to inactivate endogenous enzymes. Ubiquitin (ab7780, 1 : 50, Abcam, UK) was incubated overnight at 4°C. PBS was washed 3 times for 5 min. The secondary antibody was incubated at 37°C for 30 min. PBS was washed 3 times for 5 min. DAB was used for color development, hematoxylin was restained for 5-10 min, washed with distilled water, and PBS returned to blue. All levels of alcohol (60-100%) were dehydrated for 5 min. After removal, it was placed in xylene for 10 min and then sealed with neutral gum and observed under the microscope.

### 2.6. Immunofluorescence (IF)

The distribution of SETP6 and UBC in DU145 cells was detected by IF. The slides were removed and washed with PBS 3 times. Then, slides were fixed with 4% paraformaldehyde for 30 min, and PBS was washed 3 times for 5 min. Then, it was permeabilized with 0.5% Triton X-100 at 37°C for 30 min. After washing with PBS, 5% BSA was sealed at 37°C for 1 h, and SEPT6 (PA5-19024, 1 : 50, ThermoFisher) and UBC (ab7780, 1 : 50, Abcam, UK) were incubated overnight at 4°C. PBS was washed 3 times for 5 min. Diluted Donkey Anti-Goat IgG (H+L), FITC conjugate (SA00003-3, 1 : 100, Proteintech, UK), Donkey Anti-Rabbit IgG (H+L), and CoraLite594 conjugate (SA00013-8, 1 : 200, Proteintech, UK) were added. Then, they were incubated at 37°C for 90 min, and PBS was washed 3 times for 5 min. Then, they were dyed with DAPI (Wellbio, China) at 37°C for 10 min. The plates were sealed and observed under a fluorescence microscope.

### 2.7. Cell Counting Kit 8 (CCK-8) Assay

CCK-8 detected cell proliferation in the control group, oe-UBC group, and sh-UBC group. The cells of different groups were inoculated into 96-well plates with 1 × 10^4^ cells/well density and incubated at 37°C and 5% CO_2_ in an incubator. CCK-8 (#NU679, DOJINDO, Japan) was added 10 *μ*L/well to each well. The absorbance value at 450 nm was analyzed by a BioTek enzyme plate (MB-530, Heales, China).

### 2.8. Cell Cycle Assay

The cell suspension was collected, centrifuged to get cell precipitation, and washed with PBS 2-3 times to make the single-cell suspension, and the number of cells was adjusted to 1 × 10^6^ cells/mL. 150 *μ*L propidium iodide (PI) was added and stained at 4°C for 30 min in the dark. The PI was excited by a 488 nm argon-ion laser and received by a 630 nm pass filter. Ten thousand cells were collected through FSC/SSC scatter plot. The gating technique was used to eliminate adhesion cells and fragments, and the percentage of each cell cycle on the fluorescence histogram of PI was analyzed.

### 2.9. Clone Formation Assay

The proliferation of the control group, oe-UBC group, and sh-UBC group was detected by clone formation assay. The cells were digested with trypsin, the cell suspension was prepared, and the cells were seeded in 6-well plates at 1 × 10^5^/well. One thousand cells/2 mL were collected from each group and inoculated in a 6-well plate. The cells were cultured in an incubator at 37°C and 5% CO_2_ for 2~3 weeks, and the liquid was changed appropriately during the period. After discarding the medium, the cells were washed twice with PBS. Then, cells were fixed with 4% paraformaldehyde for 15 min, washed twice with PBS, stained with 0.5% crystal violet for 5 min, and washed with distilled water 3 times. The camera took pictures of each hole and counted the number of clones.

### 2.10. In Vivo Tumorigenesis

Nine BALB/C male nude mice were randomly divided into the NC group, sh-SEPT6 group, and oe-SEPT6 group, with 8 mice in each group. The short hairpin targeting SEPT6 (sh-SEPT6), synthesized by Sangon Biotech Co., Ltd. (Shanghai, China), was used to knock down the expression of SEPT6. To overexpress SEPT6, the SEPT6 sequences were linked to the vector LV003. SEPT6 vector was transfected into prostate cancer DU145 cells using Lipofectamine 3000 reagent according to the instructions. The right side of the mice was subcutaneously injected with 100 *μ*L PBS suspension of 1 × 10^7^ DU145 cells. The tumor volume of each group was detected at 4, 7, 11, 14, 17, 21, 24, and 28 d. After 28 days, the nude mice were sacrificed, and the tumor weight of each group was detected. qRT-PCR was used to detect the expression of SEPT6 and UBC, and WB was used to detect the expression levels of SEPT6, UBC, PCNA, and Ki67.

### 2.11. Statistical Analysis

Statistical analysis was performed using GraphPad 8.0 software (San Diego, California, USA), and three independent experimental data were expressed as mean ± standard deviation (SD). Differences between two or more groups were analyzed using the Student *t*-test or using one-way analysis of variance (ANOVA). Pearson correlation coefficient studied the correlation between SEPT6 and UBC. *P* < 0.05 was considered statistically significant.

## 3. Results

### 3.1. UBC Was Upregulated in Prostate Cancer Tissues and Cells

**Figure 1:**
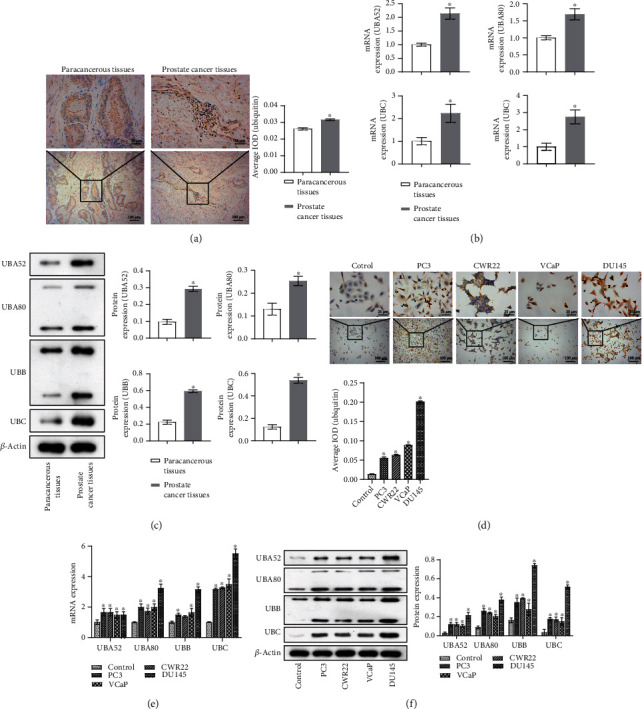
UBC was upregulated in prostate cancer tissues and cells. In prostate cancer tissues and paracancerous tissue, (a) IHC was used to detect ubiquitin-protein (UBC) expression. ^∗^*P* < 0.05 vs. paracancerous tissues; scale bar = 25 *μ*m, the magnification is 400 times; scale bar = 100 *μ*m, the magnification is 100 times. (b, c) qRT-PCR and WB were performed to detect ubiquitin-encoding gene UBA52, UBA80, UBB, and UBC expression. *P* < 0.05 vs. paracancerous tissues. In prostate cell line RWPE-1 and prostate cancer cell lines PC3, CWR22, VCAP, and DU145, (d) ICC were used to detect ubiquitin-protein expression. ^∗^*P* < 0.05 vs. the control group (prostate cell line RWPE-1); scale bar = 25 *μ*m, the magnification is 400 times; scale bar = 100 *μ*m, the magnification is 100 times. (e, f) qRT-PCR and WB detected ubiquitin-encoding gene UBA52, UBA80, UBB, and UBC expression. ^∗^*P* < 0.05 vs. the control group (prostate cell line RWPE-1).

First, IHC was used to detect ubiquitin-protein (UBC) expression in prostate cancer tissues and paracancerous tissues. UBC expression was elevated in prostate cancer tissues compared with paracancerous tissues (Figure 1(a)). WB and qRT-PCR were then performed to detect ubiquitin-encoding gene UBA52, UBA80, UBB, and UBC expression. The results revealed that UBC was highly expressed in prostate cancer tissues (Figures 1(b) and 1(c)). Then, we detected ubiquitin-protein and ubiquitin-coding gene UBA52, UBA80, UBB, and UBC expression in prostate cell line RWPE-1 (control) and prostate cancer cell lines PC3, CWR22, VCaP, and DU145. The results showed that UBC was also highly expressed in prostate cancer cells (Figures 1(d)–1(f)), consistent with our detection results in prostate cancer tissues and paracancerous tissues and highly expressed in DU145 cells. Therefore, we selected DU145 cells as the research object for subsequent experiments to explore the role of UBC in prostate cancer.

### 3.2. Overexpression of UBC Promoted Cell Survival and Proliferation

**Figure 2:**
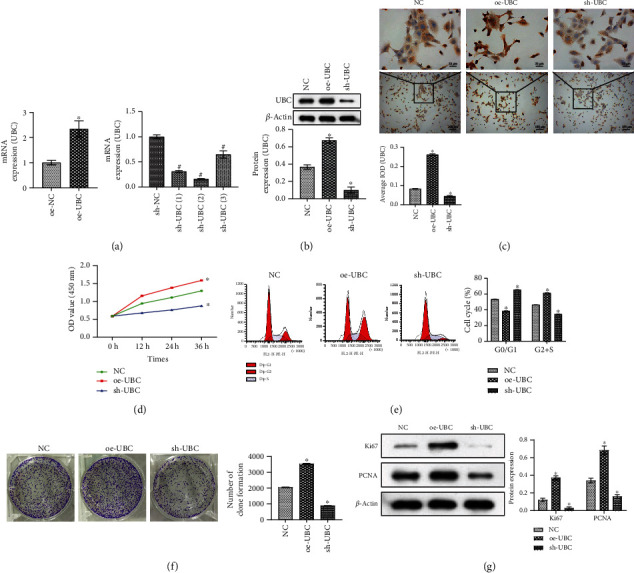
Overexpression of UBC promoted cell survival and proliferation. (a) The overexpression and knockdown efficiency of UBC was detected by qRT-PCR. (b) WB was used to detect UBC expression. (c) IHC was used to detect ubiquitin expression. (d) CCK-8 detected cell proliferation. (e) Cell cycle at 24 h was detected by flow cytometry. (f) Clonal formation assay detected cell clone number. (g) WB was performed to detect proliferation-related protein PCNA and Ki67 expressions. ^∗^*P* < 0.05 vs. the NC group; scale bar = 25 *μ*m, the magnification is 400 times; scale bar = 100 *μ*m, the magnification is 100 times.

To further study the influence of UBC on prostate cancer cells, the overexpression or knockdown of UBC expression in prostate cancer DU145 cells was divided into the oe-NC and oe-UBC groups or the sh-NC, sh-UBC (1), sh-UBC (2), and sh-UBC (3) groups. First, the knockdown efficiency of UBC and the efficiency of the overexpressed vector were verified by qRT-PCR. qRT-PCR showed that UBC expression increased in the oe-UBC group, while UBC expression in the sh-UBC (1), sh-UBC (2), and sh-UBC (3) groups was decreased. Among them, the expression of UBC was most significantly decreased in the sh-UBC (2) group, so we chose sh-UBC (2) for follow-up studies (Figure 2(a)). WB showed that sh-UBC significantly reduced UBC expression, while UBC expression increased in oe-UBC (Figure 2(b)). IHC revealed sh-UBC significantly reduced ubiquitin expression, while ubiquitin expression increased in oe-UBC (Figure 2(c)). Cell function experiments showed that compared with the NC group, cell proliferation ability and cell clone number of the oe-UBC group were increased, the G0/G1 phase was decreased, and the G2+S phase was increased (Figures 2(d)–2(f)). WB detected proliferation-related protein PCNA and Ki67 expressions. The results showed that compared with the NC group, PCNA and Ki67 expressions were increased in the oe-UBC group but decreased in the sh-UBC group (Figure 2(g)). This confirmed our hypothesis that oe-UBC promotes the proliferation of prostate cancer cells.

### 3.3. SEPT6 Mediated UBC Expression

**Figure 3:**
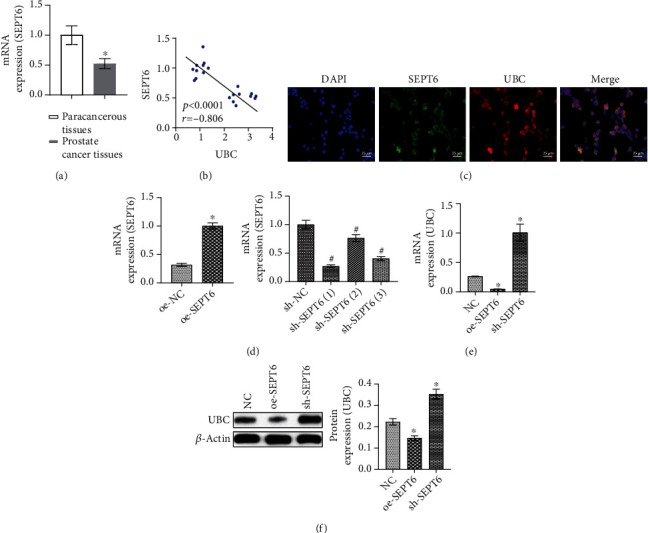
SEPT6 mediated UBC expression. (a) The expression of SEPT6 was detected by qRT-PCR in prostate cancer tissues and paracancerous tissues. ^∗^*P* < 0.05 vs. paracancerous tissues. (b) Pearson correlation coefficient analyzed the relationship between SEPT6 and UBC in prostate cancer tissues. (c) IF detected the colocation of SEPT6 and UBC in prostate cancer cells. Scale bar = 25 *μ*m, the magnification is 400 times. (d) The overexpression and knockdown efficiency of SEPT6 was detected by qRT-PCR. ^∗^*P* < 0.05 vs. the NC group. (e, f) qRT-PCR and WB detected UBC expression, respectively. ^∗^*P* < 0.05 vs. the NC group.

Previous study has found that SEPT6 might be a potential therapeutic target for prostate cancer [17]. However, there are few studies on the function of SEPT6 in prostate cancer. Therefore, we wanted to further investigate SEPT6 function in prostate cancer. We found by qRT-PCR that SEPT6 was low expressed in prostate cancer tissues compared with paracancerous tissues (Figure 3(a)). Pearson correlation coefficient analysis showed a negative correlation between SEPT6 and UBC in prostate cancer tissues (Figure 3(b)).  Figure 3(c) demonstrated the colocalization of SEPT6 and UBC in prostate cancer cells. Then, we overexpressed or knocked down the expression of SEPT6 in DU145 cells, packaged as lentivirus and transfected into cells, which were divided into the NC and oe-SEPT6 groups or the sh-NC, sh-SEPT6 (1), sh-SEPT6 (2), and sh-SEPT6 (3) groups. The knockdown and overexpression efficiency of SEPT6 was verified by qRT-PCR. qRT-PCR showed that SEPT6 expression increased in the oe-SEPT6 group, while SEPT6 expression in sh-SEPT6 (1), sh-SEPT6 (2), and sh-SEPT6 (3) groups was decreased. The sh-SEPT6 (1) group showed the most obvious decrease in SEPT6 expression, so we selected sh-SEPT6 (1) for subsequent research (Figure 3(d)). UBC expression was decreased after overexpression of SEPT6 and increased after knocking down SEPT6, indicating that UBC expression was mediated by SEPT6 (Figures 3(e) and 3(f)).

### 3.4. SEPT6 Regulated Prostate Cancer Cell Behavior through UBC

**Figure 4:**
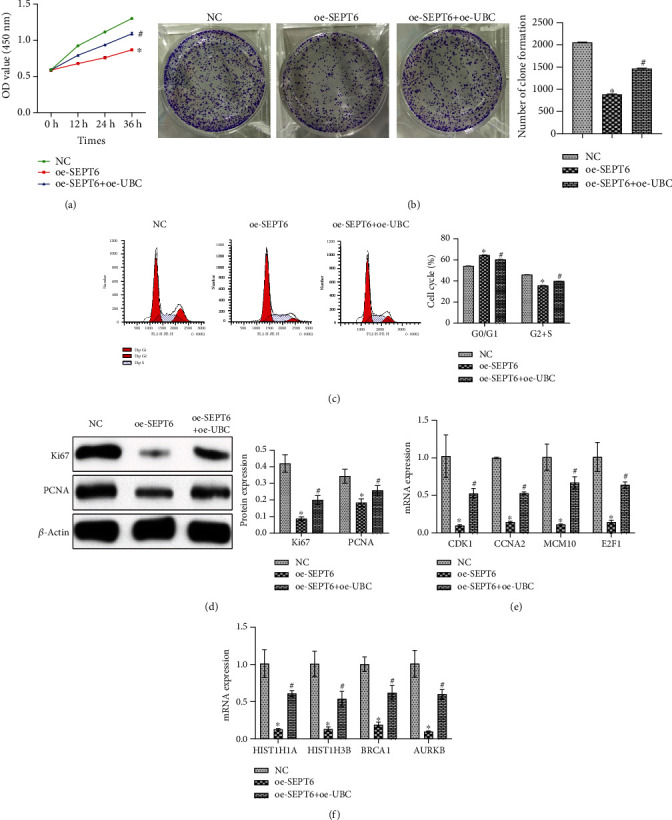
SEPT6 regulated prostate cancer cell behavior through UBC. (a) CCK-8 was used to detect cell proliferation. (b) Clonal formation assay detected cell clone number. (c) Cell cycle at 24 h was detected by flow cytometry. (d) WB was performed to detect proliferation-related protein PCNA and Ki67 expressions. (e) The expression levels of cell cycle proteins CDK1, CCNA2, MCM10, and E2F1 were detected by qRT-PCR. (f) qRT-PCR detected proliferation-related protein HIST1H1A, HIST1H3B, BRCA1, and AURKB expressions. ^∗^*P* < 0.05 vs. the NC group; ^#^*P* < 0.05 vs. the oe-SEPT6 group.

To explore the relationship between SEPT6 and UBC on prostate cancer cells, we constructed an oe-SEPT6+oe-UBC coexpression cells. They were divided into three groups: the NC (DU145 cells), oe-SEPT6, and oe-SEPT6+oe-UBC group. Cell function experiments showed that compared with the NC group, cell proliferation ability and cell clone number of the oe-SEPT6 group were decreased, the G0/G1 phase was increased, and the G2+S phase was decreased. However, cell function changes induced by oe-SEPT6 could be reversed after overexpression of UBC (Figures 4(a)–4(c)). WB detected the expression of proliferation-related proteins PCNA and Ki67. The results showed that compared with the NC group, PCNA and Ki67 expressions in the oe-SEPT6 group were reduced, and oe-UBC could reverse the decrease in the expression of PCNA and Ki67 caused by oe-SEPT6 (Figure 4(d)). Previous studies have shown that UBC reduction plays an important role in cell replication and senescence [27]. We have detected the expression levels of a series of cell cycle CDK1, CCNA2, MCM10, and E2F1 and proliferation-related proteins HIST1H1A, HIST1H3B, BRCA1, and AURKB. As shown in Figures 4(e) and 4(f), cell cycle and proliferation-related protein expressions were decreased in the oe-SEPT6 group compared with the NC group. Still, the reduced protein levels induced by oe-SEPT6 were reversed after oe-UBC. This showed that SEPT6 could regulate the behavior of prostate cancer cells through UBC.

### 3.5. SEPT6 Inhibited Tumor Growth in Nude Mice

**Figure 5:**
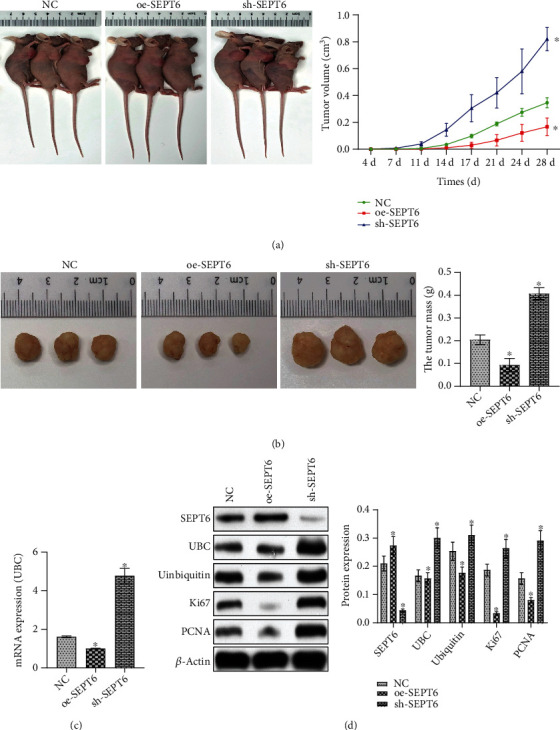
SEPT6 inhibited tumor growth in nude mice. (a) The tumor volume of each group was detected at the time points of 4, 7, 11, 14, 17, 21, 24, and 28 d. (b) Nude mice were sacrificed on day 28, and tumor mass was measured in each group. (c) UBC expressions were detected by qRT-PCR. (d) WB was performed to detect SEPT6, UBC, ubiquitin, PCNA, and Ki67 expressions. ^∗^*P* < 0.05 vs. the NC group.

To further explore the effect of SEPT6/UBC on prostate cancer, a tumor formation experiment was conducted in nude mice, and subcutaneous injection of sh-SEPT6 and oe-SEPT6 treated prostate cancer cells in nude mice. Compared with the NC group, the tumor volume and mass in the sh-SEPT6 group were significantly increased. However, the tumor volume and mass in the oe-SEPT6 group were decreased (Figures 5(a) and 5(b)). qRT-PCR showed that compared with the NC group, UBC expression in the oe-SEPT6 group was decreased, while UBC expression increased in the sh-SEPT6 group (Figure 5(c)). As shown in Figure 5(d), compared with the NC group, SEPT6 expression in the OE-SEPT6 group increased, while UBC expression decreased. In the sh-SEPT6 group, SEPT6 expression also decreased, and UBC expression increased. WB results showed that the expressions of ubiquitin and proliferation-related proteins PCNA and Ki67 were raised in the sh-SEPT6 group compared with the NC group. In the oe-SEPT6 group, ubiquitin and proliferation-related protein PCNA and Ki67 expressions were decreased. These results suggested that the SEPT6/UBC pathway could regulate the proliferation of tumor cells in vivo, thus affecting tumor progression.

## 4. Discussion

Recently, more and more groups have focused on the function of Septins in mammals. Septin can form filamentous structures and higher structures on the cortex of eukaryotic cells and bind with actin and microtubule cytoskeleton network [28]. Septins in mammals have been reported to play a key role in the cell cycle and proliferation [29]. Although Septins are essential for cytokines, apoptosis, and proliferation, their molecular mechanisms in cellular processes have not been further investigated. Our study found that SEPT6 inhibited the ubiquitin-coding gene UBC expression, affected the expression level of downstream cell proliferation-related genes, and thus affected the malignant of prostate cancer.

Posttranslational modification plays an important role in regulating the Septin-septin interaction and controlling the formation of high-order septin complexes, and such modifications include SUMOylation, acetylation, ubiquitination, and phosphorylation [30]. The covalence modification of proteins by ubiquitination is the main regulatory mechanism of protein degradation and quality control, endocytosis, vesicle transport, cell cycle control, stress response, DNA repair, growth factor signal transduction, transcription, gene silencing, and other biological fields [31]. There are four genes encoding ubiquitin in human beings, namely, UBA52, UBA80, UBB, and UBC. The amino acid sequences of ubiquitin-protein encoded by these four genes are completely the same. The only difference is that the repetition times of the ubiquitin-coding sequence in each gene are different [32, 33]. In our study, we found that UBC expression was upregulated in prostate cancer tissues and cells. At the same time, overexpression of UBC could promote cell survival and proliferation, indicating that UBC affects the function of prostate cancer cells.

SEPT6 has already been studied in cellular function. It is reported that the loss of SEPT6 could enhance hematopoietic stem cell engraftment potential [34]. LSD1-mediated SEPT6 protein activates the TGF-*β*1 pathway and regulates metastasis of non-small-cell lung cancer [35]. Notably, SEPT6 knockdown inhibited apoptosis in prostate cancer cells and promoted migration and invasion [17]. This might be due to the genetic specificity of the cell that determines the function of SEPT6. However, there are few studies on the function of SEPT6 in prostate cancer. Therefore, we wanted to further investigate SEPT6 function in prostate cancer. Proteasome and ubiquitin-mediated systems are functionally involved in many human diseases such as malignancy. It has been reported that changes in proteasome and ubiquitin gene expression are involved in the cancerous state of human kidneys [36]. However, there is little research on SEPT6 and UBC in prostate cancer. Our study found that SEPT6 was negatively correlated with UBC. Furthermore, we found the colocalization of SEPT6 and UBC in prostate cancer cells and the expression of UBC decreased when SEPT6 was overexpressed. Therefore, we speculated that SEPT6 might mediate UBC expression.

Studies have shown that targeting Septin has a strong potential to moderate harmful bystander or homeostasis cytokine-driven proliferation without affecting traditional antigen presentation-driven proliferation [37]. However, UBC regulates a series of genes that control cell cycle and proliferation, such as CDK1, CCNA2, MCM10, E2F1, BRCA1, HIST1H1A, and HIST1H3B, and induces replication of aging genes in HBM-MSCs [27]. Through cell experiments, we found that the expression levels of the cell cycle and proliferation-related proteins decreased after oe-SEPT6, but overexpression of SEPT6 and UBC could increase the protein levels. This indicates that SEPT6, as an upstream gene of UBC, could regulate the behavior of prostate cancer cells through UBC and affect cell proliferation. Through tumor formation experiments, it is found that SEPT6 could inhibit tumor growth. It shows that the SEPT6/UBC pathway could regulate the proliferation of prostate cancer cells, thereby affecting the progress of prostate cancer. However, it is still necessary to further study the role of SEPT6 and UBC in the progression of prostate cancer. In addition, there are some limitations in our study. We only used one prostate cancer cell line DU145 to study the mechanism of SEPT6 and UBC. In fact, more than two prostate cancer cell lines should be taken to study the mechanism of SEPT6 and UBC. However, due to the limitation of funds, we cannot solve this problem well at present. In the future, we will use more than two cell lines to further verify our results. On the other hand, we only investigated the effect of SEPT6 on tumorigenesis in nude mice but did not investigate the role of UBC. Due to the limitation of funds, we will construct cells that interfere with or overexpress UBC to further investigate the effect of UBC on tumorigenesis in vivo in the future.

In conclusion, SEPT6 inhibited UBC expression, thereby reducing the overall ubiquitination level, affecting the expression level of downstream cell proliferation-related genes, and thus affecting the malignancy of prostate cancer. This study provided a reference and basis for the clinical treatment and prognosis of prostate cancer in the future and provided a new target for the treatment of prostate cancer.

## Figures and Tables

**Table 1:** The primers used in this study.

Primer ID	5′-3′
UBA52-F	CGGACGCAAACATGCAGAT
UBA52-R	CGGCAAATATCAGACGCTGC
UBA80-F	AGAGACTCGGCGGTTGAAAG
UBA80-R	TCCCCGTAAGGGTTTTCACG
UBB-F	GCGCATAGAGGAGAAGGGAAA
UBB-R	AGGCTTTTCAACTGAGCCCC
UBC-F	CCGGGATTTGGGTCGCAG
UBC-R	TCACGAAGATCTGCATTGTCAAG
SEPT6-F	CACACCTACCATGACTCCCGAA
SEPT6-R	GACTCCGTTGCTGACAAGCTC
CDK1-F	AAACTACAGGTCAAGTGGTAGCC
CDK1-R	TCCTGCATAAGCACATCCTGA
CCNA2-F	ACCAGGAGAATATCAACCCGGAA
CCNA2-R	CCGGACTTCAGTACCGCCAG
MCM10-F	AAGCCTTCTCTGGTCTGCG
MCM10-R	CTGTGGCGTAACCTTCTTCAA
E2F1-F	TGCCCCACCCTCCAATCTGC
E2F1-R	CAAAACCCGGCCCAAACGTCA
HIST1H1A-F	TGGGCATTAAGAGCCTGGTAA
HIST1H1A-R	ACCCGTTGCCTTAGTTTTTGTA
HIST1H3B-F	ATGGCTCGTACTAAACAGACAGC
HIST1H3B-R	TTCCGAATCAGCAACTCGGTC
BRCA1-F	ACCTTGGAACTGTGAGAACTCT
BRCA1-R	TCTTGATCTCCCACACTGCAATA
AURKB-F	CAGTGGGACACCCGACATC
AURKB-R	GTACACGTTTCCAAACTTGCC
*β*-Actin-F	ACCCTGAAGTACCCCATCGAG
*β*-Actin-R	AGCACAGCCTGGATAGCAAC

## Data Availability

All data used to support the findings of this study are available from the corresponding author upon request.
